# Quaternized Chitosan Derivatives as Viable Antiviral Agents: Structure–Activity Correlations and Mechanisms of Action

**DOI:** 10.1021/acsami.3c01421

**Published:** 2023-04-04

**Authors:** Arun Teotia, Isabella Laurén, Sedigheh Borandeh, Jukka Seppälä

**Affiliations:** Polymer Technology, School of Chemical Engineering, Aalto University, Kemistintie 1, 02150 Espoo, Finland

**Keywords:** chitosan, double quaternization, virucidal, antimicrobial, eco-friendly

## Abstract

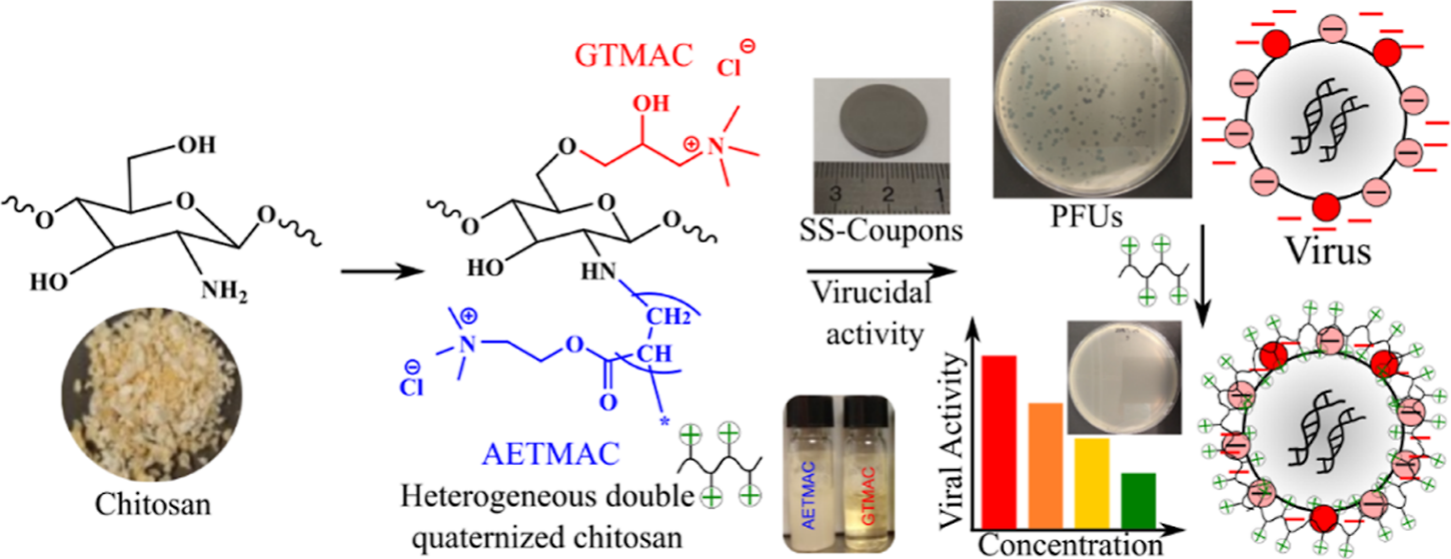

Cationic polysaccharides have demonstrated significant antimicrobial properties and have great potential in medical applications, where the antiviral activity is of great interest. As of today, alcohols and oxidizing agents are commonly used as antiviral disinfectants. However, these compounds are not environmentally safe, have short activity periods, and may cause health issues. Therefore, this study aimed to develop metal-free and environmentally friendly quaternary chitosans (QCs) with excellent long-lasting virucidal activity. To evaluate this, both single and double QCs were obtained using AETMAC ([2-(acryloyloxy)ethyl]-trimethylammonium chloride) and GTMAC (glycidyl trimethylammonium chloride) quaternary precursors. Further, this study investigated the influence of the quaternary functional group, charge density, and molecular weight (*M*_w_) on the antiviral properties of QCs. It is proposed that the higher charge density, along with the length of alkyl linkers, and hydrophobic interactions affected the antiviral activity of QCs. The findings demonstrated that heterogeneously functionalized chitosan exhibited excellent antiviral activity against both the enveloped virus φ6 and the nonenveloped viruses φX174 and MS2. These quaternized chitosan derivatives have promising potential as viable antiviral agents, as hand/surface sanitizers, or in other biomedical applications.

## 1 Introduction

From the past to present, bacteria, fungi, and viruses have caused severe infectious diseases, which have led to serious impacts on the human health and the global economy, including viruses such as the human immunodeficiency virus, the Ebola virus, MERS-CoV, and SARS-CoV-2. Certain viruses can stay in a dormant state ex vivo for a long duration until they reach their host. Virus transmission can occur through direct contact with the infected individual or indirect via bodily fluids, transmission via air, or through infected surfaces.^1^ Surfaces, including our hands, are the most important routes for viral transmission. Viruses can spread simply by touching the nasal mucosa or eye conjunctiva with contaminated hands.^2,3^ Therefore, the development of antiviral hand and surface sanitizers is necessary to limit the spread of viruses.

Due to the COVID-19 outbreak, search of materials with antiviral activity is of special interest. Currently, alcohols are the most common commercially available virucidal sanitizing agents used for the disinfection of surfaces. However, fire hazards, short contact time, and skin irritation limit their usage, as well as they have limited activity against nonenveloped viruses (e.g., Hepatitis A virus). In addition to cationic and anionic surfactants, oxidizing agents, such as sodium hypochlorite, hydrogen peroxide, and peracetic acid, are also widely used as virucidal sanitizers. Most of these agents rapidly lose their activity and often require high concentration usage (1–2%) for effective decontamination. These substances have short-lived disinfectant abilities as they require molecular disintegration for activity. Further, they are environmentally unsafe to use and toxic at higher concentrations, along with problems such as skin, mucus and eye irritation, dermatitis, and contact depigmentation. Aldehydes, such as formaldehyde, paraformaldehyde, and *ortho*-phthalaldehyde, are also used for environmental and inanimate object disinfections. Aldehydes are more effective at alkaline pH and are prohibited for direct use on skin and body tissues due to their severe toxic nature.^4^

Due to the limitations with presently available materials, there is a need for more effective materials which provide a long-lasting antimicrobial activity against both enveloped and nonenveloped viruses. Ideally, these materials can be used as environmentally friendly hand and surface disinfectants with long-lasting effects. Most viruses have a net surface charge on their bodies, which depends on the isoelectric point of the surface proteins and the pH of the virus environment. However, under normal physiological and environmental conditions, most viruses have been found to possess a net negative surface charge. This surface charge has been used to remove viruses in water treatment using coagulation agents and polyelectrolytes. Previously, we have also demonstrated that the presence of specific functional groups on the nanocellulose imparts antiviral properties.^5,6^ Recently, chitosan has been widely used in medicine.^7,8^ This linear polysaccharide, consisting of randomly distributed β-(1 → 4)-linked D-glucosamine (GlcN) and *N*-acetyl-D-glucosamine (GlcNAc) units, is commonly used in biomedical applications. However, the applications of unmodified chitosan are limited due to its poor water solubility and pH-dependent charge behavior.^9^ Surface functionalization of chitosan with cationic charged moieties considerably improves both water solubility as well as antimicrobial properties.^10,11^

The present study aims to chemically modify chitosan by incorporating carboxymethyl groups and by introducing permanent cationic charge using [2-(acryloyloxy)ethyl]-trimethylammonium chloride (AETMAC) and glycidyl trimethylammonium chloride (GTMAC) for the quaternization of chitosan. Similar compounds are previously known in the quaternization of chitosan,^12,13^ with various synthesis procedures and constellations. In this work, chitosan was quaternized in O- and N,O- positions to prepare single- and double-quaternized chitosan (SQC and DQC) in either homogeneous or heterogenous manner. Also, carboxymethyl chitosan (CMC) was quaternized and evaluated. After confirming successful quaternization, the virucidal activity of the synthesized derivatives was examined using both enveloped (φ6) and nonenveloped (φX174 and MS2) viruses at different concentrations and contact time. The main aim of this work was to investigate how the structure, the type of quaternary ammonium group, and the positive charge density can affect the antiviral activity of chitosan. Moreover, the mechanism of action of quaternary chitosan (QC) and its ability to bind to negatively charged viruses were evaluated. Quaternized polysaccharides can potentially serve as biobased agents with virucidal/antimicrobial activity for environment friendly hand and surface sanitizers.

## 2 Experimental Section

### 2.1 Materials

Chitosan [degree of deacetylation (DDA) ≥75%; purity: 99%; CAS no. 9012-76-4] was purchased from TCI (Japan). AETMAC, GTMAC, and ammonium persulfate were purchased from Sigma-Aldrich (USA). Sodium hydrogen carbonate (NaHCO_3_) and benzaldehyde (C_7_H_6_O) were obtained from Merck (Germany). Cetyl trimethyl ammonium bromide, calcium chloride, magnesium sulphate, lecithin, peptone, Tween-80, and sodium thiosulphate were purchased from Sigma-Aldrich (USA). Bacteriophage Phi-X174 (cat. no. 124425) and its corresponding host *Escherichia coli*-C (cat. no. 124400) were purchased from Carolina Biological (Burlington, USA). Bacteriophage Phi-6 (cat. no. 21518) and MS2 (cat. no. 13767) along with their corresponding host organisms *Pseudomonas syringae* (cat. no. 21482) and *E. coli* (Migula 1895, cat. no. 5695) (*E. coli*-M) were purchased from DSMZ-German Collection of Microorganisms and Cell Cultures (Braunschweig, Germany). Luria-Bertini (LB) broth was purchased from Condalab (Madrid, Spain).

### 2.2 Preparation of Quaternized Carboxymethyl Chitosan

*O*-carboxymethyl chitosan (*O*-CMC) was prepared by suspending 10.0 g (62.12 mmol) of chitosan in 100 mL of 50% NaOH and stirred overnight at −18 °C. After heating to room temperature, 20 mL of isopropanol was added to the suspension, followed by gradually adding 35.0 g of chloroacetic acid and 100 mL of isopropanol over a period of 30 min. Then, the temperature was increased to 30 °C, and the suspension was stirred for 4 h. The reaction was terminated by adding 200 mL of ethanol, and the product was neutralized with HCl and later freeze-dried.

This was followed by quaternization of *O*-CMC in the *N*-position with either GTMAC or AETMAC. *O*-CMC was quaternized with GTMAC according to the previously described protocol by Spinelli et al.,^13^ with a ratio of 3.0 g of *O*-CMC (13.7 mmol) and 6.0 g of GTMAC (39.6 mmol). *O*-CMC quaternized with AETMAC was prepared as follows: 1.0 g (4.6 mmol) of *O*-CMC was dissolved in 100 mL of 2.0 wt % acetic acid at 80 °C. Then, 1.0 g of ammonium persulfate, as the initiator, and 2.0 mL (11.7 mmol) of AETMAC were gradually added to the solution, followed by stirring for 3 h at 80 °C under a N_2_ atmosphere. After cooling to room temperature, the obtained product was precipitated by acetone and further washed with methanol to remove unreacted AETMAC monomers and byproducts. The quaternized products were freeze-dried.

### 2.3 Preparation of Single and Double Quaternary Ammonium Chitosan

In this study, quaternary compounds AETMAC and GTMAC were introduced to chitosan in either O- or N,O-position, generating single and double QCs (SQC and DQC, respectively). To successfully introduce quaternary compounds in the *O*-position solely (SQC), the amine of the chitosan was protected by benzaldehyde, which was later removed to enable successful double quaternization in both O- and N-positions. Schiff base chitosan (*N*-benzylidene chitosan) was prepared according to the protocol described by Fu et al.^14^ using 2.0 g (12.4 mmol) of chitosan and 13.14 g (124.0 mmol) of benzaldehyde. Successful modification of the amine with benzaldehyde was confirmed with FTIR, where the characteristic peak of C=N vibration was seen at 1640 cm^–1^ (Figure S1).

**Scheme 1:**
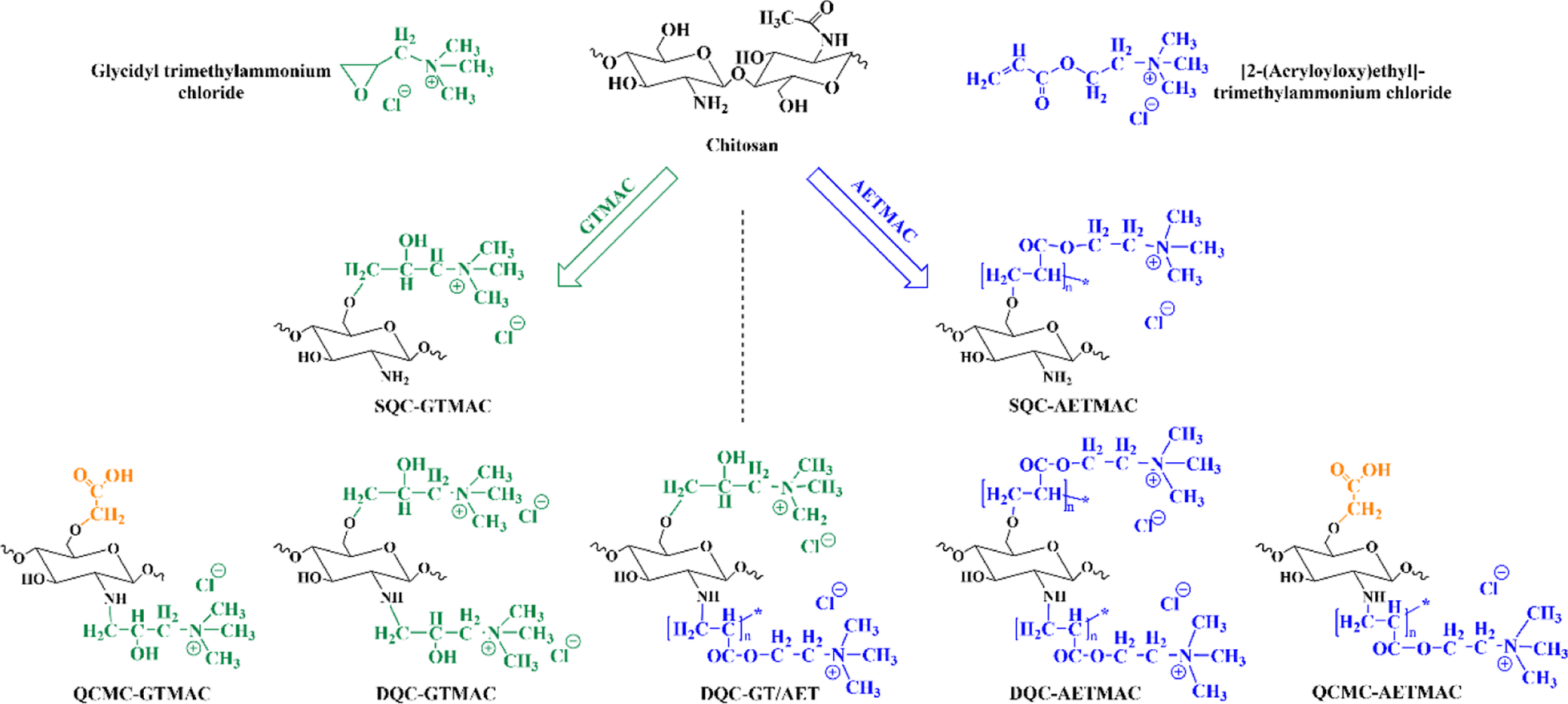
Schematic Representation of Different GTMAC and AETMAC Functionalized QC Families

The obtained Schiff base chitosan was then quaternized in the O-position with either GTMAC or AETMAC, in a similar manner as previously described in the Preparation of Quaternized Carboxymethyl Chitosan. Here, we used a ratio of 1.5 g (6.0 mmol) of Schiff base chitosan to 5.6 g (37.3 mmol) of GTMAC, and 1.0 g (4.0 mmol) of Schiff base chitosan to 2.0 mL (11.7 mmol) of AETMAC. After quaternization, the protected amine was deprotonated by suspending the product in 0.1 M HCl in ethanol overnight, generating SQC-GTMAC and SQC-AETMAC. To prepare DQC, the quaternization process was repeated as mentioned before using 1.5 g (5.4 mmol) of SQC-GTMAC or 1.0 g (3.2 mmol) of SQC-AETMAC as starting materials. The successful introduction of quaternary compounds was confirmed with Fourier transform infrared spectra (FT-IR) and ^1^H NMR. Schematic representations of all structures, with their respective given names, are given in Scheme 1.

### 2.4 Structural Characterization

#### 2.4.1 FT-IR Spectroscopy

FT-IR spectra were evaluated using a Spectrum-Two ATR-FT-IR Spectrometer (PerkinElmer, UK). The powder samples were scanned in a range between 4000 and 500 cm^–1^, with a resolution of 4 cm^–1^ and 32 accumulations.

#### 2.4.2 ^1^H and ^13^C NMR Spectroscopy

^1^H NMR analyses were determined with a Bruker Avance III 400 spectrometer (Bruker, USA), and ^13^C NMR was acquired using a Bruker Avance NEO 600 (Bruker, USA) spectrometer. The synthesized derivatives were dissolved in D_2_O, whereas pure chitosan was dissolved in 2.0 wt % DCl/D_2_O.

#### 2.4.3 Zeta Potential

The zeta potential was analyzed with a Zeta sizer Nano ZS 90 (Malvern PANanalytical, UK) in the pH range between 3 and 10.5. The samples were dissolved in water, and the pH was adjusted with HCl or NaOH.

#### 2.4.4 Molecular Weight Estimation

The molecular weights of different samples were estimated using gel permeation chromatography (GPC) using a PLaquagel-OH MIXED-M column (×2) (Varian Inc., USA) for efficient separation. The samples were dissolved overnight in elution buffer [0.1 M CH_3_COOH +0.1 M NaCl (pH-2.8)] at a concentration of 2.0 mg/mL and then filtered using a 0.22 μm filter before analysis. The separation was performed using a 1260 Infinity system (Agilent Technologies, USA) at the following conditions: flow rate of 0.7 mL/min, injection volume 100 μL, UV_280_, refractive index (RI), LS-15/90°, with a runtime of 45 min per sample. Pullulan standards (*M*_w_ 110 000 and 200 000 g/mol) were used for system calibration.

#### 2.4.5 Rheological and Solubility Analyses

Aqueous solution viscosity of different polymers was measured under constant shear conditions at a shear rate (γ) sweep from 0.1 to 100 S^–1^ using an Anton Paar Physica MCR 301 rheometer. A cone and plate geometry with a diameter of 50 mm, cone angle α = 0.983°, and truncation gap of 50 μm was used. Samples were analyzed at 20 °C using a Peltier heated/cooled hood and bottom plate, precautions were taken to minimize solvent evaporation. Kinematic and intrinsic viscosities of low concentration solutions (<2.0 gm/L) of different materials were also calculated at 20 °C using a Ubbelohde viscometer (Schott Gerate, Germany) after applying appropriate correction factors.

Aqueous solution turbidity (% transmission at 660 nm) was analyzed to evaluate the aqueous solubility of different materials and the influence of temperature on solubility. The samples were completely dissolved in Milli-Q type-1 water (0.04 μS/cm) and gently centrifuged to remove any undissolved particles before measurements. Measurements were carried out under constant stirring using a Cary 5000 UV–vis–NIR spectrometer equipped with Peltier heated/cooled glass cuvettes. Milli-Q water was used as a reference control.

### 2.5 Propagation of Phages and Their Host

Hosts of the phages *E. coli*-C and *P. syringae* were cultured on LB broth and LB-agar plates at 37 and 28 °C, respectively, whereas *E. coli*-M was cultured in Medium-271 (ATCC) with streptomycin (2.0 mg/L). Growth characteristics of the organisms in LB medium were investigated to evaluate the suitability of using LB for growing host organisms. To propagate test organisms, 100 μL of phage stock culture (φ6, φX174 and MS2) was mixed with 100 μL of host organisms, that had been cultured overnight, in 800 μL of LB broth supplemented with 20 mM Ca^2+^ and Mg^2+^ ions (LB^+/+^). The mixture was added to 3 mL of LB-agar (0.3% w/v) and cultured using a double-layer agar (DLA) method. The plates were incubated overnight for phage propagation. The following day, the plates were flooded with 5 mL of LB^+/+^ medium and placed in a shaker at 50 rpm at 28 °C for 4 h. The broth from all plates was collected, pooled, and centrifuged at 4500 rpm for 30 min. Then, the supernatant was filtered through a 0.22 μm syringe filter and stored at 4 °C. No further phage purification was performed before using phages for the virucidal activity assay. The purified phages were titrated by the DLA-plaque forming unit (PFU) assay to calculate the PFUs/mL present in the phage stock solution.

### 2.6 Virucidal Assessment

For the virucidal activity analysis, well-established biosafe [Biosafety level 1 (BSL-1)] bacteriophage surrogates for more pathogenic mammalian viruses were selected as test organisms.

#### 2.6.1 Selection of Potential Surrogates

For the selection of a potential surrogate, the viability of the virus on a stainless-steel (SS) surface and the effect of drying on its viability were considered as critical components. Only a virus demonstrating no significant drop in the viability (PFUs) after a 60 min drying process was used in the assay. We settled on the enveloped virus Phi6 (φ6) and the nonenveloped viruses PhiX174 (φX174) and MS2 as test organisms. These are also considered as biosafe test surrogates by US-Environmental protection agency (US-EPA) for the Ebola virus.^15,16^ Using both enveloped and nonenveloped viruses as test organisms enabled us to test the extent of the antiviral spectrum of the QC derivatives.

#### 2.6.2 Virucidal Activity Analysis

The virucidal activity analyses of different virucidal agents were estimated according to ASTM Standards,^17^ DVV, and US-EPA guidelines, with appropriate modifications.^18^

#### 2.6.3 Inoculation of a Virus to the Surface or in a Suspension

Tests were carried out using SS coupons as carriers to quantitatively evaluate the virucidal disinfection activity of the compounds on nonporous surfaces as dispersions in bovine serum albumin (BSA) solution for the suspension assay. For deposition of the virus on SS carrier discs (dia. 15 mm), aqueous BSA solution (3 mg/mL) containing 20 mM Ca^2+^ and Mg^2+^ ions, sterilized by 0.22 μm filtration, was used as the loading solution. A 10-fold dilution of 100 μL of the viral stock solution dispersed in 900 μL of loading solution, making a final volume of 1000 μL, represented a 10^–1^ dilution. Appropriate viral dilutions were used to seed the carriers with >6 average log_10_ PFU per carrier, to achieve 4 or higher average log reduction (average log_10_ PFU) of viral load. The carriers were placed in 55 mm sterile Petri dishes, and the surface of the carriers was inoculated with 100 μL of appropriate dilution of the viral stock solution (>10^9^ PFU/mL) in the loading solution, leading to a deposition of >10^6^ virus particles (>6 average log_10_ PFU) per carrier. The carrier coupons were either dried under laminar air flow (dried conditions) or covered with a nonvent lid to prevent drying (nondried conditions). The solution was dried on the surface until visibly dry. In all cases, the samples were dried only for a maximum of 60 min under laminar air flow, and the discs were used for testing within 30 min after the drying step. During each test, the nondried and nontreated group was used as a recovery control for calculating the average log_10_ reduction in the viral titre under different treatment conditions in the carrier-based assay.

#### 2.6.4 Virus Exposure to Test Materials

The test materials (100 μL), dissolved in sterile deionized H_2_O, were applied to the SS disc, covering the whole surface area. The discs were incubated for a contact time of 10 min. Then, they were flooded with 400 μL of ice-cold neutralization solution: peptone (1 g/L), lecithin (0.7 g/L), Tween-80 (5.0 g/L), sodium thiosulphate (1 g/L), and an appropriate quantity of ice-cold broth to make the final volume to 1000 μL, leading to a 10-fold dilution (10^–1^). The discs were incubated for 10 min followed by thorough pipetting to recover the viruses from the surface. The recovered solution was collected and stored on ice (maximum of 2 h) until further processing. The SS discs that were deposited with a virus-containing loading solution and not subjected to the drying step or treatment with test compounds were used as a negative control for the drying effect and the recovery of the viruses from the discs. Similarly, viral deposition subjected only to the drying step and not to the test compounds were used as postdrying recovery controls. For direct suspension assays, 100 μL of virus dilution was mixed with 100 μL of double concentration (2×) solution of the test substance in a 2.0 mL centrifuge tube, mixed well, and incubated for appropriate duration, followed by neutralization and dilution as mentioned above.

The recovered viruses were further given 10-fold serial dilutions (10^–2^,10^–3^,10^–4^,10^–5^...) by diluting 100 μL of the recovered solution in 900 μL of respective broth medium to obtain individually separated countable number of plaques on the DLA plate. Host organisms (100 μL) that had been cultured overnight in LB^+/+^ were added to 900 μL of the test dilution and were incubated for 5 min. Subsequently, the inoculum was added to 3 mL of LB-agar (0.3%) present at 50 °C in a 15 mL capped falcon tube, mixed thoroughly, and poured onto a Petri plate for the DLA assay. The plates were incubated at an appropriate temperature for 24 h before testing for plaque formation. If required, the plates were further incubated for 24 h for revised plaque counting. All tests for conditions mentioned were performed in duplicate each time and repeated at least twice to calculate average the log_10_ reduction values. A compound was considered virucidal/phagicidal if the average reduction of >4 log_10_ values were obtained in PFU values under test conditions.

### 2.7 Transmission Electron Microscopy

For transmission electron microscopy (TEM) micrographs, the harvested virus particles were purified via polyethylene glycol (PEG) precipitation. Briefly, the lysates were allowed to precipitate overnight at 4 °C in 1:1 PEG-NaCl buffer [PEG-8000 (20 wt %) + NaCl (2.5 M)] followed by centrifugation. The pellets were redissolved in an appropriate volume of SM buffer [sodium chloride (100 mM), Tris (50 mM), and MgSO_4_ (8 mM)]. For imaging, the viral particles (1 μL) were dispersed in 0.22 μL of filtered Milli-Q water (4 μL) and then mixed with 4 μL of uranyl formate (1% w/v). The viral particles were deposited on formvar-carbon copper grids and imaged using a FEI Tecnai-12 microscope operating at 120 KV of accelerating voltage and equipped with a Gatan US1000 CCD camera. For Cryo-TEM, nonpurified phage was directly used from the lysate to prevent any virus inactivation or change in morphology due to the purification process. The images were taken without any staining. The viruses were deposited on lacey carbon copper grids, and imaging was performed using a JEOL-3200FSC cryo-TEM microscope operating at 300 KV.

### 2.8 QC-Virus Interaction Mechanisms

To investigate the interactions between the QCs and the virus particles, rescue studies were performed. After a 10 min treatment with AETMAC and GTMAC derivatives in the suspension (nondried) state, the QC was inactivated using neutralization buffer, and MS2 phages were serially diluted (10^–2^, 10^–3^, 10^–4^...) and subjected to the DLA assay. PFUs were observed for both AETMAC and GTMAC derivatives at different dilutions to investigate viral killing or inhibition of host interaction. Cryo-TEM imaging was performed to observe the structural changes in the virus envelope using φ6. Here, 100 μL of 10^–1^ dilution of φ6 viral particles were treated with 100 μL of DQC-GT/AET (100 mg/mL) for 10 min, followed by centrifugation at 1000*g* for 10 min to remove the debris. The supernatant was serially 10^–6^-fold diluted and stored on ice until cryo-TEM analysis. The viral dilution (4 μL) was loaded on the plasma-cleaned lacey carbon copper grid and processed using Vitrobot for the TEM analysis. Nontreated φ6 viral particles were used as the control for evaluating the structural changes in the phage envelope post treatment.

## 3 Results and Discussion

As previously mentioned, under certain environmental conditions (pH > pI), the viral proteins carry a negative charge, providing a net negative charge to the virus particle.^19^ However, the protonation of chitosan amine groups only occurs at lower pH, which generates a positive charge on the compound responsible for enhanced solubility and antimicrobial properties. Quaternization will introduce a permanent positive charge to the chitosan structure, improving its solubility and antimicrobial activity at a wide pH range.^20^

### 3.1 Preparation and Characterization of QCs

In this study, quaternary groups were introduced onto the chitosan’s structure using AETMAC and GTMAC (Scheme 1) to enhance the positive charge and to improve its water solubility and virucidal activity. The aim of using two different functionalized groups was to find the most appropriate quaternary compound leading to high virucidal activity. The structures were characterized with FT-IR, NMR, GPC, and zeta potential to confirm successful quaternization and thus a permanent positive charge over a wide pH range.

#### 3.1.1 FT-IR Spectroscopy

**Figure 1:**
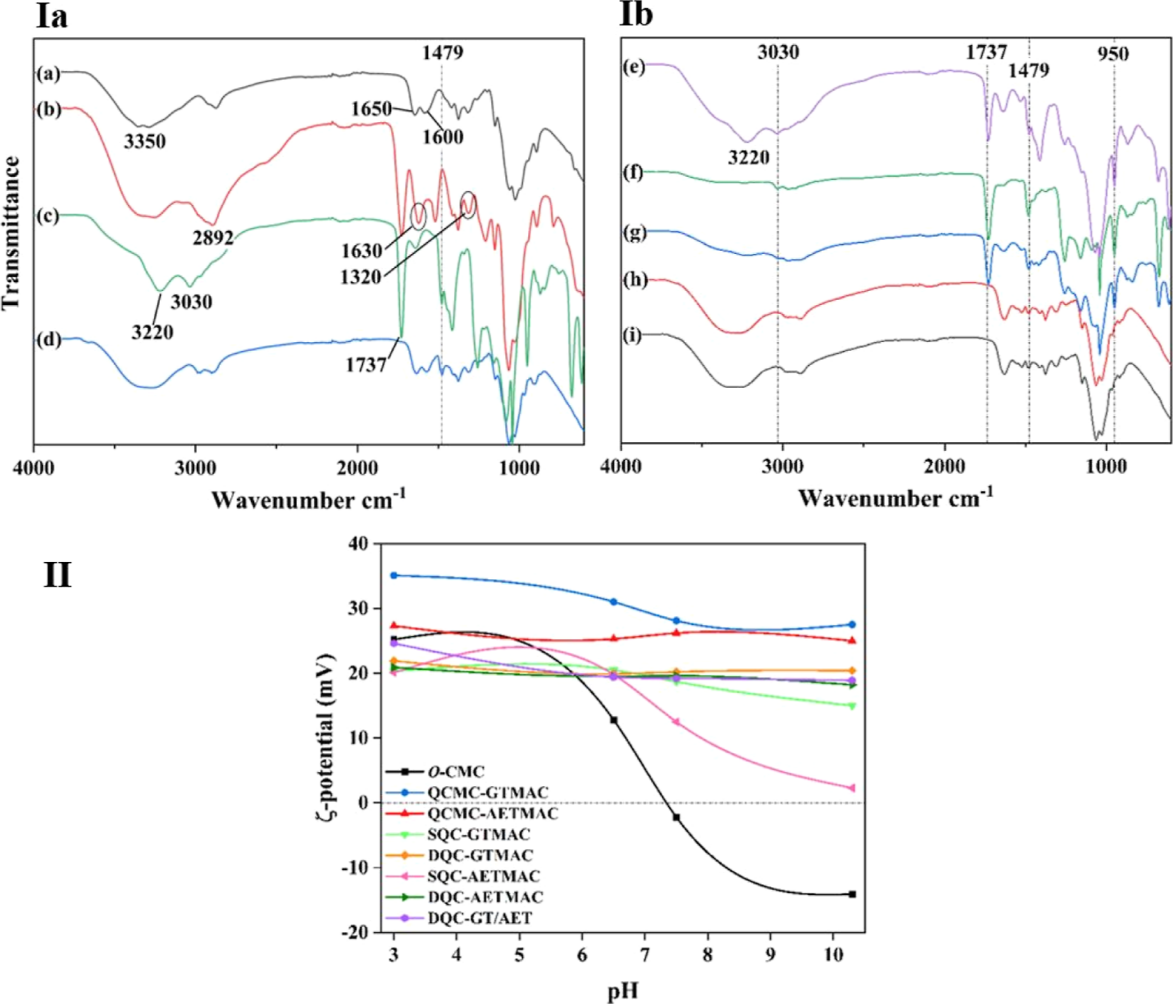
(Ia) FT-IR spectra of (a) chitosan, (b) *O*-CMC, (c) QCMC-AETMAC and (d) QCMC-GTMAC. (Ib) FT-IR spectra of (e) DQC-GT/AET, (f) DQC-AETMAC, (g) SQC-AETMAC, (h) DQC-GTMAC, and (i) SQC-GTMAC. (II) Zeta potential of chitosan derivatives between pH 3 and 10.

To confirm successful introduction of quaternary compounds to chitosan, FT-IR analysis was used. The spectra of chitosan, *O*-CMC, and their quaternized derivatives are shown in Figure 1I. Characteristic bands of chitosan were found at around 3350 cm^–1^, associated with the N–H and the O–H vibrations of amino and hydroxyl groups. The C=O stretching vibration of primary amide groups was located at 1650 cm^–1^, as well as the N–H bending and C–N stretching vibrations of secondary amide groups were located at 1600 cm^–1^. Carboxymethylation of chitosan resulted in additional bands at 1737 and 1630 cm^–1^ corresponding to N–H and carbonyl stretching vibrations, respectively, and a stronger C–O stretching vibration at 1320 cm^–1^.

When comparing quaternized derivatives to unmodified chitosan, disappearance of the characteristic N–H and C–N vibration band at 1600 cm^–1^ was noticed, which can be attributed to the deformation of the primary amine N–H vibration at the secondary amide band, implying that the −NH_2_ group of the chitosan was reacted. The appearance of a new peak for the QCs at approximately 1479 cm^–1^ was attributed to the asymmetrical stretching of the methyl groups of the quaternary ammonium salt. For the QCs quaternized with AETMAC, the two absorption bands at approximately 1737 and 950 cm^–1^ could be assigned to the C=O stretching vibrations of the ester groups and the C–N vibration of the quaternary ammonium groups, respectively, suggesting successful introduction of AETMAC to chitosan. Furthermore, the broadband ascribed to the hydroxyl groups and primary amine groups found at approximately 3350 cm^–1^ disappeared after quaternization, and two new peaks at approximately 3220 and 3030 cm^–1^ appeared, likely ascribed to the secondary amine. This confirms the quaternization of chitosan, and the results are consistent with the previous literature.^21−23^

#### 3.1.2 Zeta Potential Measurement

The cationic charge is an important aspect regarding the antimicrobial/flocculation properties of chitosan. However, chitosan only possesses a positive charge in acidic conditions due the protonation of the amine. In neutral and alkaline conditions, the amine loses its protonation and thus its remarkable antimicrobial properties. To overcome these limitations, a permanent positive charge can be introduced to the chitosan by quaternization and hence exhibits excellent antimicrobial activity over a broader pH range.^20,24^ As seen in Figure 1II, the nonquaternized amino groups of *O*-CMC and SQCs became deprotonated at neutral and alkaline pH, and hence the potential switches toward a more negative charge. Differently, the DQCs and CMC-QCs show a permanent positive charge also at alkaline pH, indicating the successful quaternization of the amine.

#### 3.1.3 ^1^H and ^13^C NMR Spectroscopies

**Figure 2:**
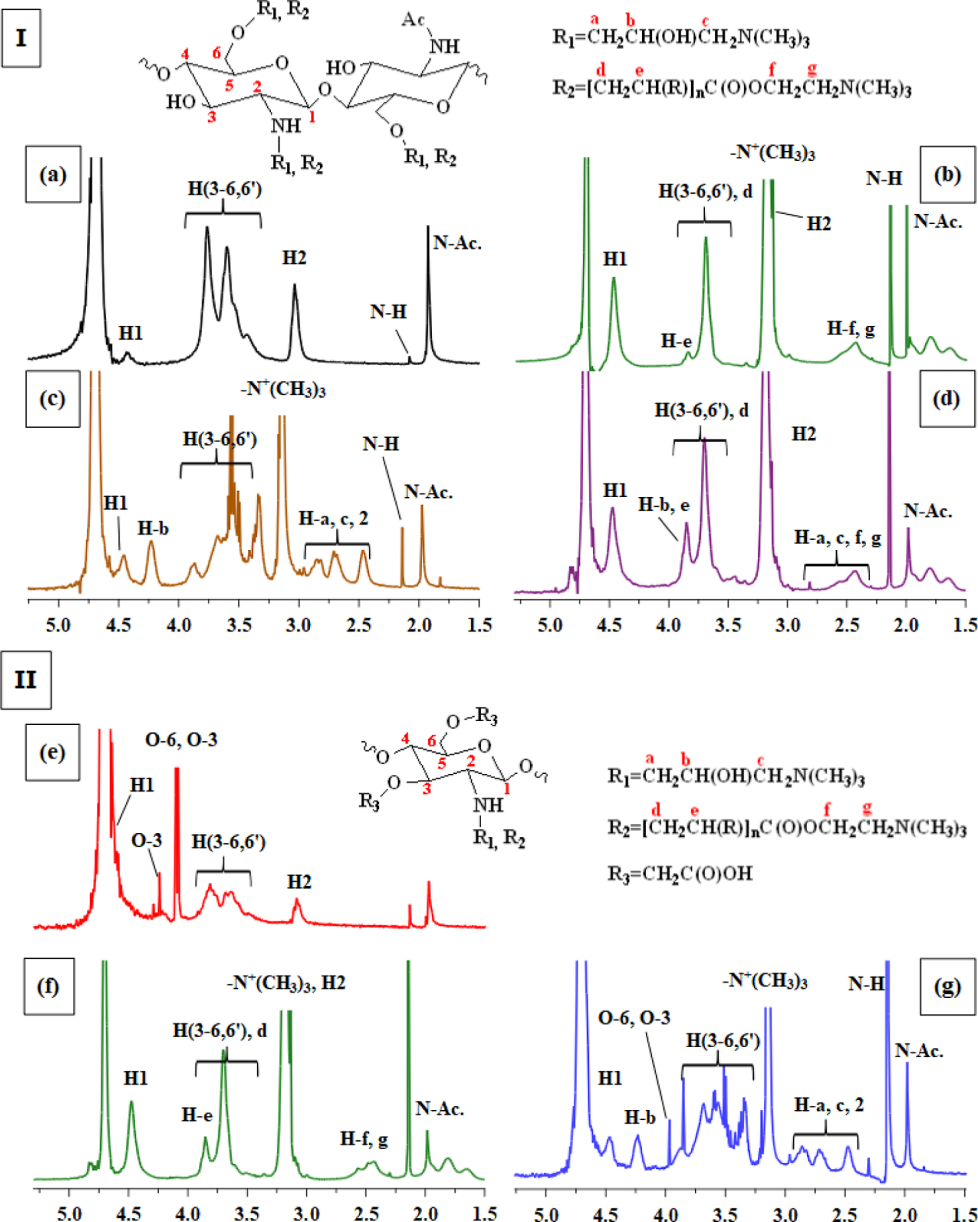
^1^H NMR spectra of (I) quaternized chitosan derivatives and (II) quaternized CMC derivatives. Here, the spectra are (a) chitosan, (b) DQC-AETMAC, (c) DQC-GTMAC, (d) DQC-GT/AET, (e) *O-*CMC, (f) QCMC-AETMAC, and (g) QCMC-GTMAC. R_1_ represents GTMAC; R_2_ represents AETMAC; R_3_ is a carboxymethyl group.

The successful introduction of AETMAC and GTMAC to chitosan and *O*-CMC was determined and characterized with ^1^H NMR; see Figure 2. Characteristic peaks assigned to the chitosan skeleton can be found at approximately δ = 1.95 [*N*-acetyl], δ = 2.10 [N–H], δ = 3.06 [H2-GlcN], δ = 3.45–3.80 [H2-GlcNAc, H3–H6, H6′], and δ = 4.5–4.6 ppm [H1-GlcNAc] and overlapping at δ = 4.85 ppm [H1-GlcN].^12,25^ The DDA of chitosan was calculated according to eq 1, where [CH_3_] is the integral corresponding to the acetyl peak of the GlcNAc units found at approximately 2.00 ppm, and [H2-GlcNAc, H3–H6, H6′] corresponds to the pyranosyl protons found approximately at 3.3–4.0 ppm.^25,26^
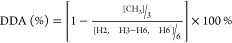
1

According to eq 1, the calculated average DDA of pure chitosan was ≈76.1%, which corresponds to the manufacturer’s reported value of DDA ≥75%.

Further, the signals corresponding to the protons of *O*-CMC (either 3- or 6- substituted *O*-CMC) occurred in the region between 4.0 and 4.4 ppm. The resonance found at δ = 4.10 ppm was ascribed to three protons from C-6 (two protons) and C-3 (one proton) positions, whereas the peak found at δ = 4.25 ppm was ascribed to one proton from C-3. The total fraction of carboxymethylation (F) was calculated according to methods described by Hjerde et al.^27^ using the following equations
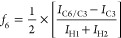
2
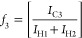
3
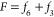
4where *f*_6_ and *f*_3_ are the fractions of the carboxymethylation in positions C-6 and C-3, respectively, and *F* is the total average degree of substitution varying between 0 and 2. *I*_x_ are the intensities of the peaks ascribed to H1 (GlcNAc, δ = 4.65 ppm), H2 (GlcN, δ = 3.10 ppm), C-6/C-3 carboxymethyl protons (δ = 4.10 ppm), and C-3 carboxymethyl proton (δ = 4.25); see Figure 2. The calculated values were *f*_6_ = 1.42 and *f*_3_ = 0.26, resulting in a total degree of carboxymethylation of 1.68. Further, the hydroxyl group at C-6 has higher reactivity compared to the hydroxyl group located at C-3, which was confirmed with eqs 2 and 3. This was also confirmed with ^13^C NMR (Figure S3), where the intensity at δ = 62.81 ppm (C-6) was significantly larger than that of C-3 (δ = 73.17 ppm). In addition, N-substitution is favored at higher temperatures, and therefore, the amino groups of chitosan should not be carboxymethylated in this reaction, which was confirmed by the absence of a peak at approximately δ = 3.25 ppm.^28,29^

Furthermore, regarding the QCs quaternized with AETMAC, signals found at δ = 2.01 [*N*-acetyl], δ = 2.14 [N–H], δ = 2.35–2.4 [H9, H10], δ = 3.05–3.18 [H2-GlcN, −N^+^(CH_3_)_3_ group], δ = 3.36 [three methyl groups of ammonium], δ = 3.60–3.80 [H2-GlcNAc, H3–H6, H6’], δ = 3.70 [H5; N^+^–CH_2_–CH_2_–O– stretch], and δ = 3.85 [H8]; δ = 4.48 [H1-GlcNAc] further confirmed the introduction of the quaternary compound AETMAC to the chitosan skeleton.^12,23^ In a similar manner, peaks assigned to chitosan quaternized with GTMAC were found approximately at δ = 1.97 [*N*-acetyl], δ = 2.14 [N–H], δ = 2.48 [H2-GlcN], δ = 2.70 [H9], δ = 2.83 [H7], δ = 3.15 [−N^+^(CH_3_)_3_ group], δ = 3.3–3.9 [H2-GlcNAc, H3–H6, H6′], δ = 4.23 [H8], and δ = 4.46 [H1-GlcNAc], and the result is consistent with previous reports.^30^ The peak attributed to the solvent was found at δ = 4.70 ppm. ^1^H NMR spectra of SQCs are seen in Figure S2.

The degree of quaternization (dQ) of the quaternized derivatives was calculated according to the integrals of the −N^+^(CH_3_)_3_ peak found approximately at 3.15–3.18 ppm, and the pyranosyl protons [H2-GlcNAc, H3–H6, H6′] were found approximately between 3.6 and 4.2 ppm (eq 5).^31^ The obtained dQ values are reported in Table 1 and the integrals are found in Figure S2. Furthermore, the C-6 position is in favor of quaternization in SQC and DQC compared to the hydroxyl group found in the C-3 position, due to higher reactivity. ^13^C NMR spectra of selected compounds are found in Figure S3, where the downfield shift in the C-6 position (δ = 58.75 ppm in chitosan) to approximately δ = 62 ppm confirms functionalization. Further, a higher intensity peak in the C-6 position compared to C-3 indicates higher reactivity. This is in accordance with previous literature.^27,32,33^
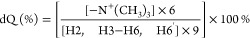
5

**Table 1:** dQ, d*n*/d*c*, and average *M*_w_ of Quaternized Chitosan Derivatives^a^

sample	dQ	d*n*/d*c* [mL/g]	*M*_w_ [g/mol]
chitosan	ND	0.174	59 264
SQC-AETMAC	1.11	0.134	5 056
DQC-AETMAC	1.76	0.144	7 300
QCMC-AETMAC	1.51	ND	ND
SQC-GTMAC	0.41	0.183	34 730
DQC-GTMAC	1.01	0.176	25 022
QCMC-GTMAC	0.67	ND	ND
DQC-GT/AET	1.3	0.142	12 854

a The dQ was calculated according to eq 5. ND = No data available.

#### 3.1.4 Molecular Weight Estimation

**Figure 3:**
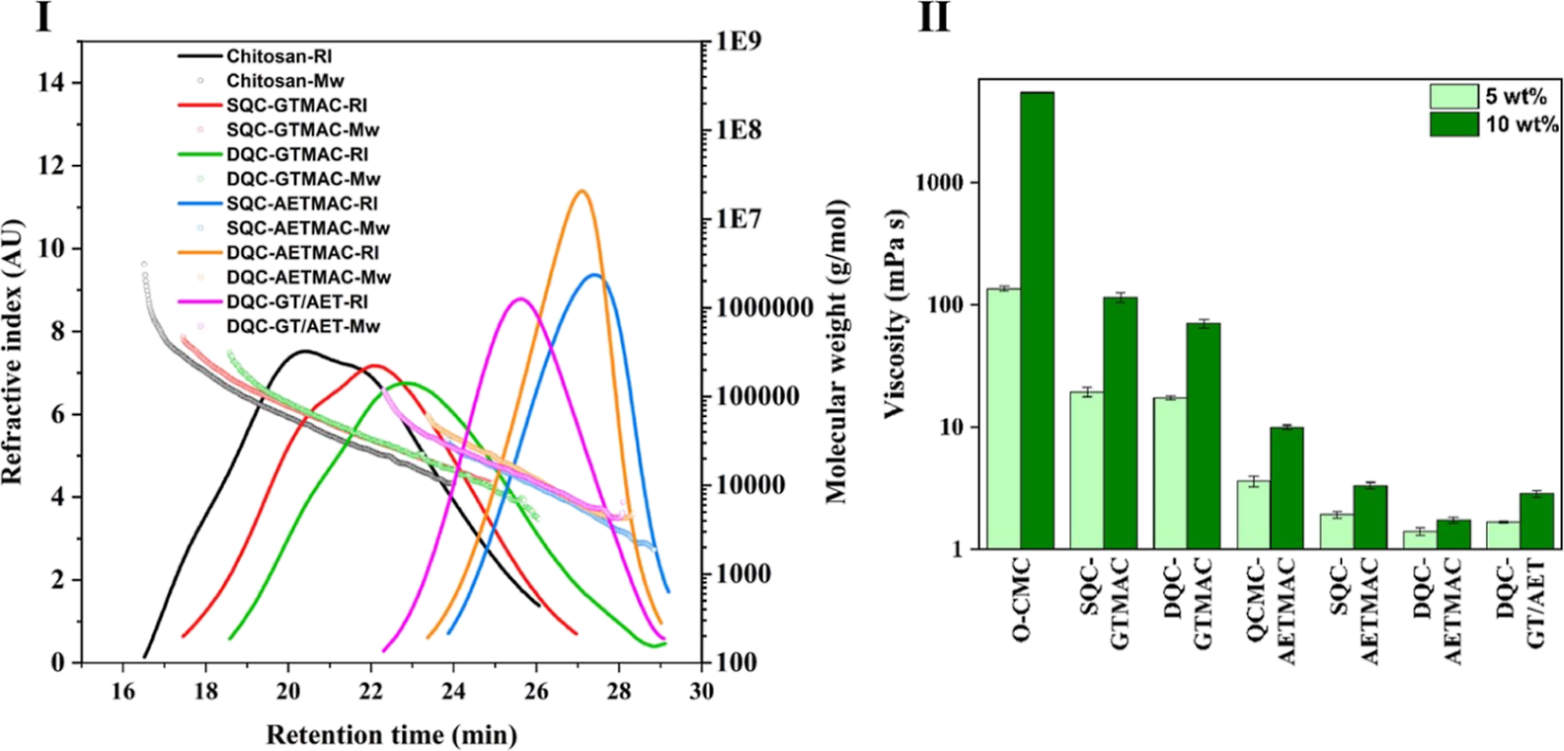
(I) RI and the obtained molecular weight (*M*_w_) of chitosan and QCs. (II) Solution behavior of chitosan derivatives: average viscosity (log_10_) of *O*-CMC and QCs at concentrations of 5 and 10 wt %. Chitosan and QCMC-GTMAC led to formations of very viscous gels and were therefore excluded due to processing difficulties.

The molecular weights (*M*_w_) of chitosan and its derivatives were evaluated with GPC. Native chitosan demonstrated the shortest elution time with a broader elution peak (Figure 3I), and this was closely followed by SQC-GTMAC and DQC-GTMAC, respectively. In comparison to chitosan and GTMAC derivatives, AETMAC derivatives demonstrated a late elution which was approx. 1.5 times longer than the chitosan elution time. Interestingly, the bifunctional DQC-GT/AET-derivative eluted midway between GTMAC and AETMAC derivatives. When this was correlated with the d*n*/d*c* values and the *M*_w_ of various derivatives, a decrease in *M*_w_ was observed with increasing dQ of the polymers (Table 1). The decrease in *M*_w_ was smaller in GTMAC derivatives compared to AETMAC derivatives. Further, the double-quaternized DQC-GTMAC had lower *M*_w_ compared to its single-quaternized counterpart, SQC-GTMAC. This decrease in *M*_w_ can be correlated with a longer reaction time, and exposure to harsh reaction conditions and elevated temperatures may lead to chain scission and consequently a decrease in the *M*_w_.^34^ Furthermore, the AETMAC derivatives had much lower *M*_w_ compared to GTMAC derivatives (Figure 3I). DQC-AETMAC had slightly higher *M*_w_ compared to SQC-AETMAC, as expected. Here, the vinyl groups of the AETMAC derivatives built new chains branching from the parent chitosan chain during polymerization compared to direct functional modification with GTMAC derivatives. This is in correlation with DQC-AETMAC having slightly higher molecular weight compared to SQC-AETMAC, even though the former also had higher dQ. These differences in the charge densities on the polymeric chains may also be influencing their aqueous solution behavior, along with temperature response toward solubility and observed phase separation.

#### 3.1.5 Solubility and Rheological Analysis

As discussed earlier, low pH solubility of chitosan limits its application, whereas the QCs demonstrated a high level of solubility. However, GTMAC derivatives lead to high viscosity solutions compared to AETMAC derivatives; see Figure 3II. The viscosity of the solution governed the workable concentrations of the compound. High-viscosity solutions were difficult to pipette, apply, or spread on the SS coupons, as well as mix uniformly with the viral suspensions. QCMC-GTMAC led to the formation of highly viscous gels, posing handling problems to conduct the tests, and therefore it was not included in the virucidal activity assays. Both SQC- and DQC-AETMAC efficiently solubilized/dispersed in H_2_O, leading to turbid solutions without significantly enhancing the solution viscosity. Comparatively, QCMC-AETMAC solutions demonstrated a lower turbidity, which can be attributed to its higher solubility due to the presence of carboxymethyl groups. DQC-GT/AET also gave rise to transparent yellowish low viscosity solutions with no turbidity, which also can be attributed to the presence of the more hydrophilic GTMAC as the additional functional group on the molecule, enhancing the overall solubility. As QCs demonstrate a contact-based inhibition, solution viscosity might influence the antiviral properties of the compounds. The kinematic viscosities at low concentrations of GTMAC-QCs were also close to the kinematic viscosity of chitosan (Figure S3). However, the AETMAC derivatives had very low kinematic viscosity. This demonstrates differences in solution behavior of these materials, which can be correlated with the differences in the molecular weight, where the molecular weight of a molecule directly correlates with the solution viscosity and the nature of the functional groups.

**Figure 4:**
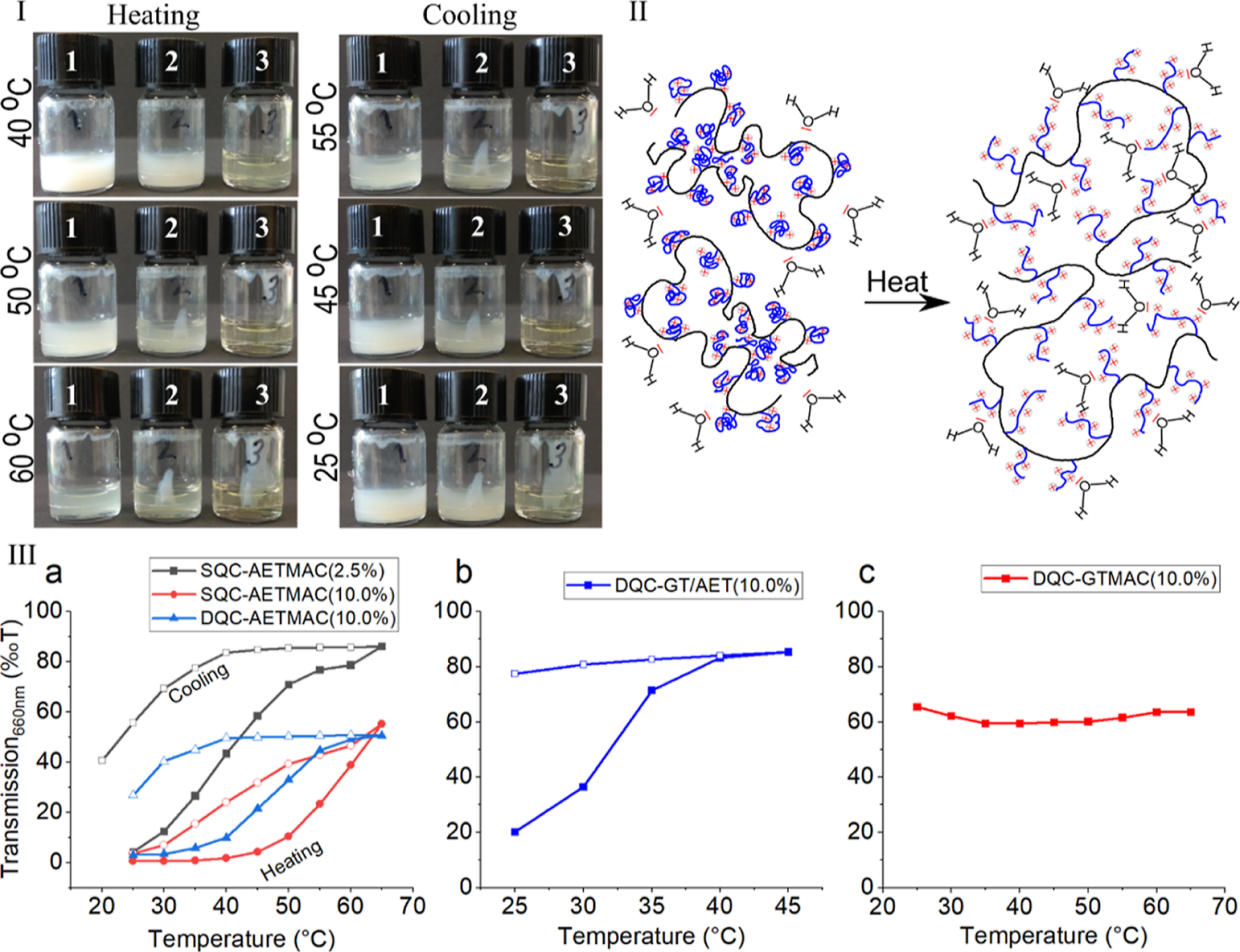
Temperature-induced solubility transition behavior of QC derivatives. (I) Images showing temperature-induced change in aqueous solubility of (1) SQC-AETMAC, (2) DQC-AETMAC, and (3) DQC-GT/AET, showing cloud points at around 40 °C for DQC-AETMAC and at around 50 °C for SQC-AETMAC and a reversion in solubility on cooling to 25 °C. (II) Schematic representing the change in molecular interaction between QC chains and water molecules at lower temperatures and chain uncoiling inducing enhanced solubility at elevated temperatures. (III) Hysteresis curves representing changes in solution light transmission with an increase (close boxes) and decrease (open boxes) in temperature.

Furthermore, the AETMAC derivatives demonstrated temperature-dependent aqueous solubility; see Figure 4. These derivatives had higher solution turbidity, with a temperature-dependent transition behavior due to the cloud point phenomenon at lower temperatures. This could be because of chain uncoiling at elevated temperatures, leading to enhanced interactions between water molecules, hence increased solubility. DQC-AETMAC had a cloud point at around 40 °C, while SQC-AETMAC had at a slightly higher temperature, around 50 °C (Figure 4IIIa); the transition occurred within a couple of minutes of temperature change. An enhanced solubilization (rapid transition) along with delayed precipitation was observed at lower concentrations (2.5%), demonstrating a hysteresis in transition. Both showed a reversion in solubility on cooling to 25 °C, suggesting the presence of chain entanglement-induced inter- and/or intrachain hydrophobic interactions at lower temperatures, leading to phase separation. DQC-GT/AET also demonstrated enhanced solubilization with an increase in temperature; however, the reversion in solubility was occurring slow over longer time periods (Figure 4IIIb). Here, the GTMAC functional groups might also be influencing the overall solubility and stabilizing interactions with water molecules, leading to enhanced solubility and a delay in temperature response. This can also be observed in Figure 4IIIc where DQC-GTMAC demonstrates no significant effect of temperature on solubility.

### 3.2 Phage Propagation and Imaging

**Figure 5:**
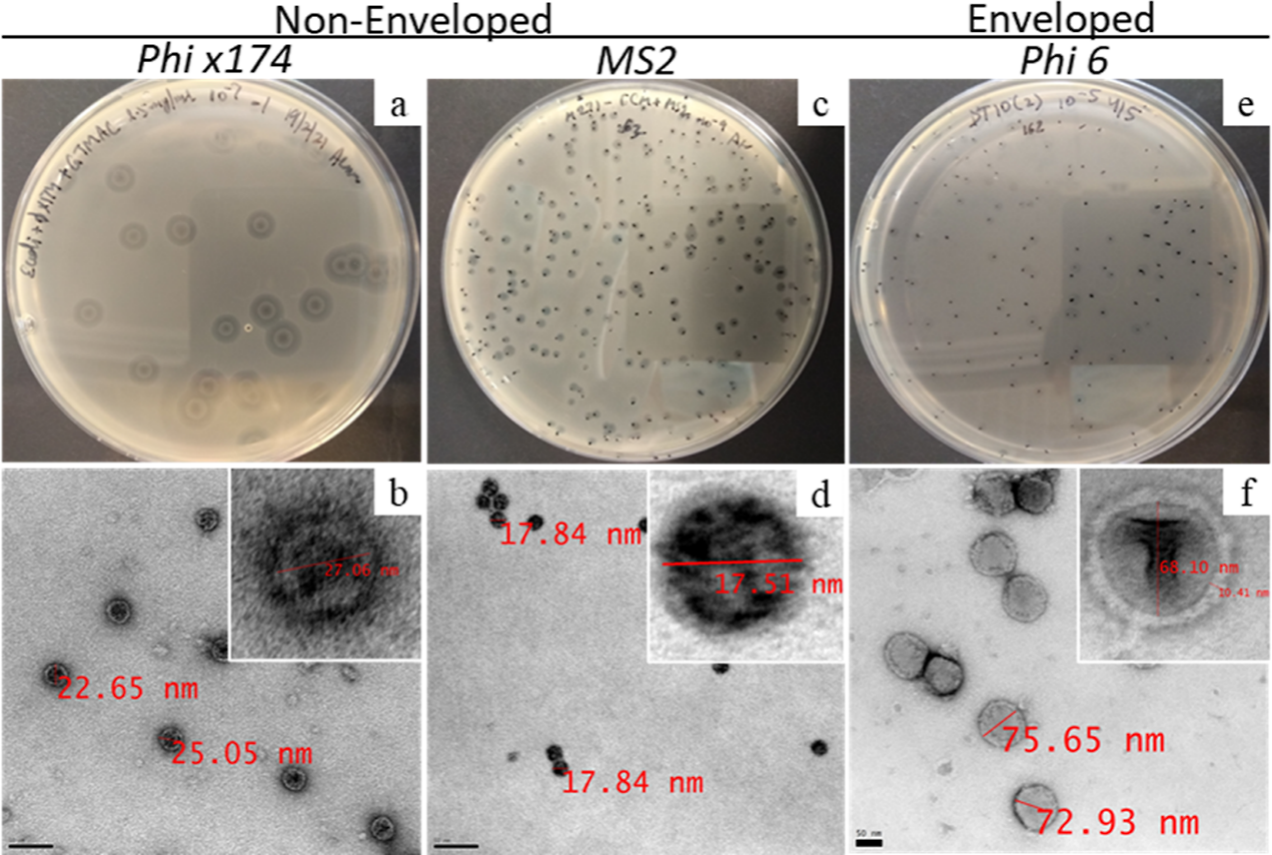
Photographs and TEM micrographs of different viruses studied. Images representing the plaque characteristics of different viruses in a lawn of their respective host organisms, and TEM micrographs of the respective virus, where (a,b) φX174 and (c,d) MS2 are nonenveloped viruses and (e,f) represents φ6, an enveloped virus with an envelope of ∼10 nm thickness (scale bar 50 nm).

The viruses were collected after successfully propagating into the lawns of their respective hosts, leading to high titre (PFUs) values. The harvested viral stocks had titres of 10^9^ to 10^10^ PFU/mL of the virus particles. Viruses demonstrated clear plaque formation on the lawn in DLA assays. The plaque size and morphology differed among viruses (Figure 5), with φX174 showing large plaques with a “bull’s eye” morphology, whereas MS2 and φ6 had smaller plaques. Morphological characterization of purified phages using TEM demonstrates nonenveloped phages being smaller in size, φX174 (∼25 nm) and MS2 (∼17.8 nm), whereas φ6 was larger (∼75–80 nm) with the presence of a distinct envelope surrounding the nucleocapsid.

### 3.3 Effect of Drying

The effect of drying on the viability or on the recovery of the virus (average log_10_ reduction) was elucidated prior to conducting the virucidal activity evaluation. Additionally, nondried control replicates were included in every test. The average reduction in the recovery/viability after the drying step was 0.26% for φX174, 0.1% for MS2, and 1.28% for φ6. The viral suspension deposited on SS coupons were used as negative controls for both drying and recovery. The controls for drying were always included in the tests and used to calculate the reduction in phage titres (average log_10_ reductions) for different treatments. The virucidal values were calculated and normalized accordingly.

### 3.4 Virucidal Activity

#### 3.4.1 Treatment with Virucidal Compounds

**Figure 6:**
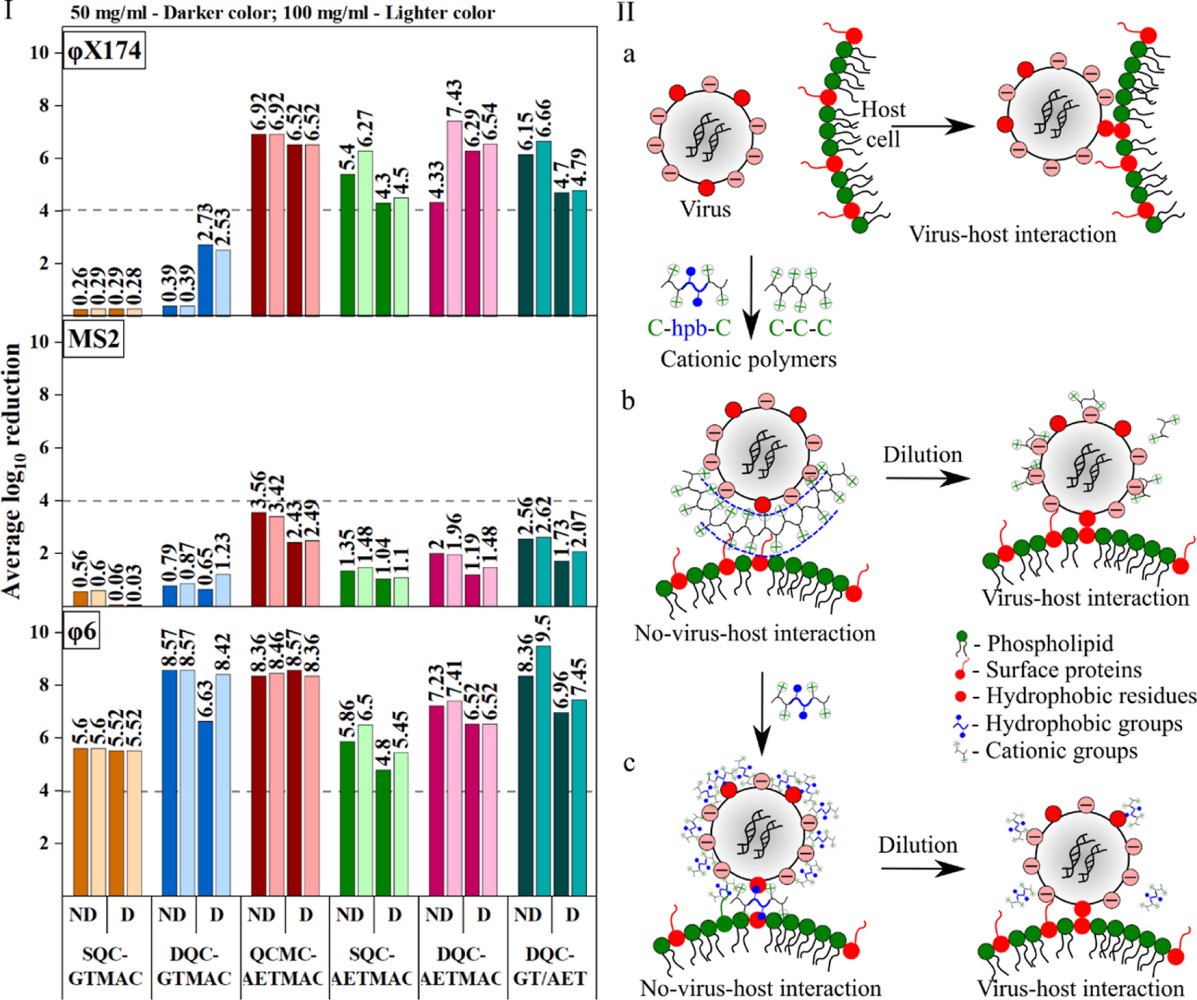
Virucidal activity of different compounds against φX174, MS2, and φ6 viruses. (I) Average log_10_ reduction of the viruses when exposed to different concentrations of the test compounds (50 or 100 mg/mL) for 10 min under both dried (D) and nondried (ND) conditions. The compound was considered antiviral if the average logarithmic reduction was greater than 4. (IIa) Schematic representing the role of hydrophobic–hydrophobic interactions between the viral spike proteins and the host surface receptor necessary for host–virus interaction. (IIb) Cationic chitosan (C–C) blocking the host–virus interaction due to charge–charge interactions with viral particles and (IIc) by blocking the hydrophobic–hydrophobic virus–host interactions due to the presence of hydrophobic (hpb) interactions.

Based on the preliminary evaluation, the virucidal activity of compounds were determined for a 10 min contact time at 50 and 100 mg/mL working concentrations under both dried (D) and nondried (ND) conditions. A compound was considered virucidal/phagicidal if >4 average log_10_ reduction in PFUs was observed under the test conditions (Figure 6 and Table S1). Here, the enveloped phage φ6 was much more susceptible to neutralization by the QC compounds in comparison to the nonenveloped viruses (φX174, MS2), demonstrating different levels of susceptibility to the QCs. MS2 demonstrated the lowest inactivation by both compound series. Here, a higher removal activity was demonstrated by AETMAC derivatives compared to the GTMAC derivatives; however, it was significantly lower than the threshold value of >4 average log_10_ reduction mark (Figure 6I). The dQ as well as single or double quaternization were other factors influencing the activity. AETMAC derivatives demonstrated an overall higher activity compared to GTMAC derivatives, and DQCs showed higher activity than SQCs. Furthermore, the anti-viral behavior was also dependent on the concentration, dried or nondried conditions, and exposure time (data not shown).

Interestingly, the two heterogenous structures containing either both GTMAC and AETMAC (DQC-GT/AET) or a carboxymethyl group (QCMC-AETMAC) demonstrated higher activity against all viruses tested (Figure 6I) compared to the homogenous structures. Here again, φ6 demonstrated the highest reduction followed by φX174; MS2 demonstrated the lowest reduction under any conditions. Furthermore, the viral reduction was higher under nondried conditions compared to dried conditions for all samples. This might be due to slower solubilization of viruses embedded in the dried BSA matrix, lower mixing, and interactions between polymers and viruses (Figure 6II), which might not be the case under nondried (suspended) conditions. This also suggests a contact-based inhibition mechanism of inactivation. This goes in accordance with the cationic nature of the compounds, suggesting a charge-based interaction with the viral particles and either destabilization of the capsid proteins responsible for host binding or direct blocking of the virus–host interactions.

#### 3.4.2 QC-Virus Interaction Mechanisms

As previously reported, it is assumed that by increasing the dQ and the positive charge density, the antimicrobial activity of QCs should increase.^35,36^ In this study, although GTMAC derivatives and AETMAC derivatives had an almost equal dQ, AETMAC-QCs outperformed GTMAC-QCs regarding the antiviral activity. The correlation between AETMAC derivatives and higher antiviral activity can be corroborated with their lower *M*_w_, smaller size, and higher dQ compared to GTMAC derivatives. However, several factors contribute toward the overall activity and influence their interaction mechanisms and activities. According to the literature, the hydrophilicity/hydrophobicity and chain length of the substituent/linker can also affect the antimicrobial activity of QCs. It is proposed that in addition to the charge–charge interactions, the hydrophobic interactions between the QCs and the hydrophobic surface groups of the viral surface proteins can be proposed mechanisms of action of QCs. Moreover, the linker length of the substituents on the QCs has been demonstrated to influence their antimicrobial activities.^37−40^ Accordingly, by comparing the structures in AETMAC derivatives to those of GTMAC derivatives (Scheme 1), it can be concluded that small *M*_w_ derivatives might interact with viral particles more efficiently, destabilize the surface proteins, or inhibit host–virus interactions by forming surface-corona structures. Additionally, longer linkers might also take part in the hydrophobic interactions with the surface proteins, as observed in the phase separation with SQC- and DQC-AETMAC derivatives (Figure 4), endowing DQC-AETMAC with higher virucidal activity.

**Figure 7:**
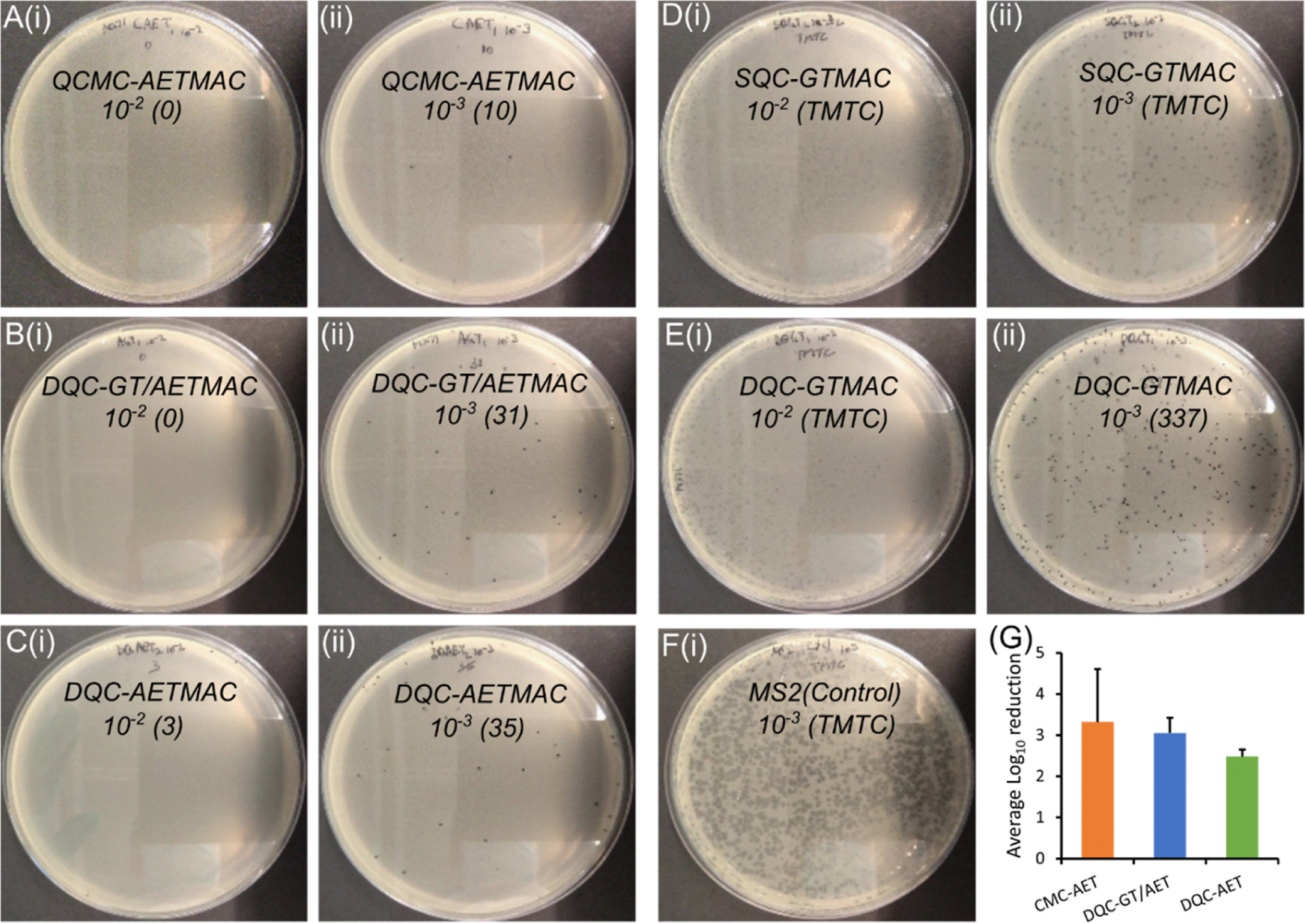
Reduction in the PFU density of MS2 after treatment with QCs. The phages were treated with different (A–C) AETMAC and (B,D,E) GTMAC derivatives for 10 min in the suspension state, demonstrating the level of inactivation. (i) Photographs showing PFUs at lower serial dilution (10^–2^) with corresponding PFU values in brackets, demonstrating very low or nil PFUs. (ii) Photographs showing PFUs at higher serial dilution (10^–3^) with higher PFU numbers, demonstrating re-emergence of infective virus particles. The phenomenon was less prominent (not measurable) with GTMAC derivatives solely (D,E). (F) Viral load in the test control at 10^–3^ dilution. (G) Graph showing the numerical average log_10_ reduction values by different AETMAC derivatives for higher dilutions (A–C). [*n* ≥ 3; error bars = standard deviation (SD); TMTC = too many to count].

Furthermore, while conducting rescue studies during the virucidal activity analysis for different AETMAC derivatives, a re-emergence of PFUs was frequently observed at lower serial dilutions (10^–3^); see Figure 7. Owing to their lower activity and the presence of large number of PFUs, a similar phenomenon was not observed with the GTMAC derivatives. This phenomenon was more clearly detected with the more resilient MS2 phage, giving us further insight into the mechanisms of viral inhibition. First, the emergence of a larger number of PFUs with GTMAC derivatives, compared to AETMAC derivatives, clearly demonstrates higher activity of AETMAC derivatives. On the DLA assay, a higher number of PFUs was observed for both SQC- and DQC-GTMAC even at lower (10^–2^, 10^–3^) dilutions, with a proportionate decrease at higher dilutions. For AETMAC derivatives treated with MS2, no PFUs were observed at 10^–2^ dilution, but at higher dilutions (10^–3^), a re-emergence of PFUs was observed (Figure 7). A re-emergence of PFUs at higher dilutions suggests a QC-virus interaction responsible for viral inactivation. A coat around viral particles by QCs might disable the virus–host interaction (Figure 6IIb). At higher dilutions, some of these interactions might get destabilized, enabling viral escape and host infection (emergence of PFUs).

Viral-host surface protein interactions are important for successful infection. The φX174 virus interacts via the hydrophobic sequences (AAFLG, Figure S4) in protein-F and the hydrophobic domains in the *E. coli* cell surface lipopolysaccharides.^41^ Similarly, MS2 and φ6 interact with respective hosts by specifically interacting with pili-specific proteins. Here, the MS2 surface maturation (Mat) protein interacts via hydrophobic (Val15, Phe31, Leu33, Phe92, and Phe94) pockets with the first five amino acid sequences (AGSSG, Figure S4) of tra-A proteins of F-pili via hydrophobic–hydrophobic interactions.^42^ Similarly, φ6 uses envelope spike protein P3 for initial interaction with type IV pili for adhesion and protein P6 for fusion with the host via hydrophobic interactions.^43,44^ Further, it was observed that purified *P. syringae* pili binds with P3, decreasing φ6 infectivity by 85% PFUs, which reverts on higher dilutions.^45^

**Figure 8:**
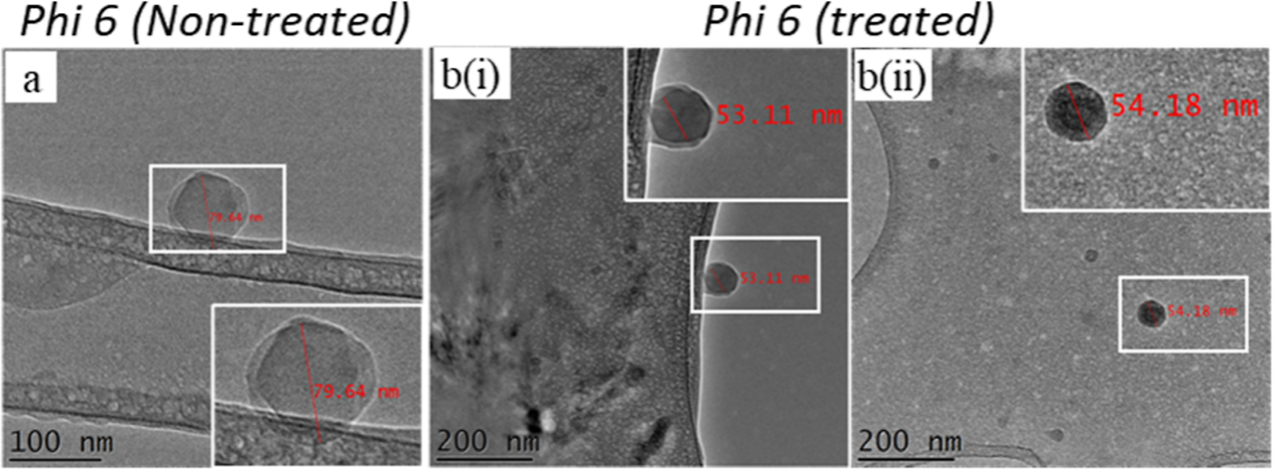
TEM photographs displaying the changes in virus (φ6) structure (a) before and (b) after treatment with DQC-GT/AET, demonstrating a change in the structure/size of the virus.

It is well established that virus–host interactions are enabled by specific interactions between the viral surface spike proteins and host receptor proteins.^46^ φX174, MS2, and φ6 interact with the respective hosts by specific cell surface proteins via the hydrophobic sequences.^41−44^ An elimination of PFUs at higher concentrations and a re-emergence on dilution (Figure 7) suggest inhibition of host–virus interactions at higher concentrations as the mechanism of action. Further, cryo-TEM of φ6 viral particles treated with DQC-GT/AETMAC also demonstrates a distortion of round viral envelope from ∼80 nm (nontreated) and decreasing to ∼54 nm after treatment (Figure 8).

This change in size demonstrates an interaction between the compound and the viral envelope proteins, destabilizing the envelope/structural proteins and inhibiting the virus–host interactions. Our findings suggest that in addition to the charge interactions, a smaller *M*_w_, the presence of hydrophobic interactions, and structural protein destabilization influence the antiviral activity of quaternized chitosan.

## 4 Conclusions

FT-IR, NMR, GPC, and zeta potential measurements demonstrated successful introduction of quaternary functional groups, imparting a permanent charge to chitosan over a wide pH range. Further, the evaluation of antiviral activity of AETMAC and GTMAC derivatives concluded that heterogeneously functionalized chitosan (DQC-GT/AET and QCMC-AETMAC) showed higher antiviral activity compared to homogenously functionalized chitosan. Further, AETMAC derivatives showed overall significantly higher antiviral activity against both nonenveloped viruses φX174 and MS2 and enveloped virus φ6 compared to GTMAC derivatives. It is suggested that the antiviral behavior of QCs is dependent on their *M*_w_ and charge density. Further, the inhibition of specific interaction between viral-host surface proteins and the destabilization of viral surface proteins by interactions between viral capsid/envelope and the functional QCs are responsible for their overall activity. This research concludes that the presence of longer alkyl linkers in AETMAC cationic moieties significantly improves the antiviral activity of QC, especially against the enveloped virus φ6. This research proposes that quaternized chitosan, with high antiviral activity, can be a biosafe alternative to commercially available antiviral agents for various applications.

## Supporting Information Available

FT-IR spectra of chitosan and Schiff base chitosan, ^1^H NMR spectrographs of chitosan and different derivatives showing peak integrals, ^13^C NMR spectrographs of certain chitosan derivatives, kinematic viscosity calculation for low-concentration solutions, spike protein sequences of different viruses, and table of viral average log_10_ reduction values for different bacteriophages (PDF)
