# Supplementary material for: Quaternized Chitosan Derivatives as Viable Antiviral Agents: Structure–Activity Correlations and Mechanisms of Action
Source: ACS Appl Mater Interfaces. 2023 Apr 4;15(15):18707–19. doi: 10.1021/acsami.3c01421 (PMC10119858; doi:10.1021/acsami.3c01421)
Supplement: Supplementary file 1 — am3c01421_si_001.pdf [file am3c01421_si_001.pdf]

Supporting Information

Quaternized chitosan derivatives as viable antiviral agents: structure-activity correlations and mechanisms of action

Arun Teotia†, Isabella Laurén†, Sedigheh Borandeh, Jukka Seppälä*

Polymer Technology, School of Chemical Engineering, Aalto University, Kemistintie 1, 02150 Espoo, Finland

† These authors had the same contribution.

* Corresponding author: jukka.seppala@aalto.fi

Figures

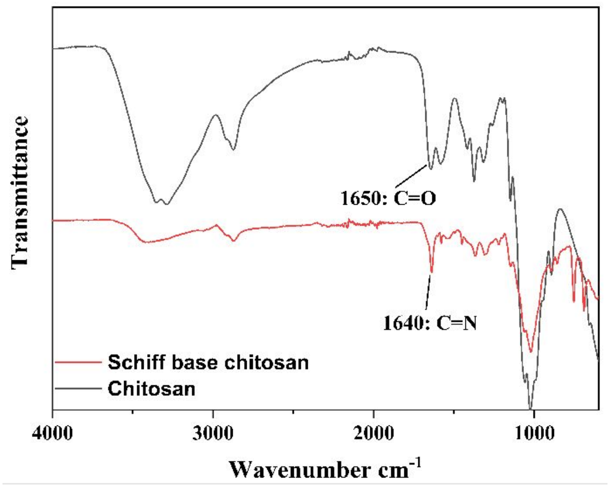

Figure S1 FTIR spectra of chitosan and Schiff base chitosan. Most peaks of the Schiff base chitosan were weakened due to the addition of a Schiff base. The C=N peak at 1640 cm^{-1} confirms the modification of the amine.

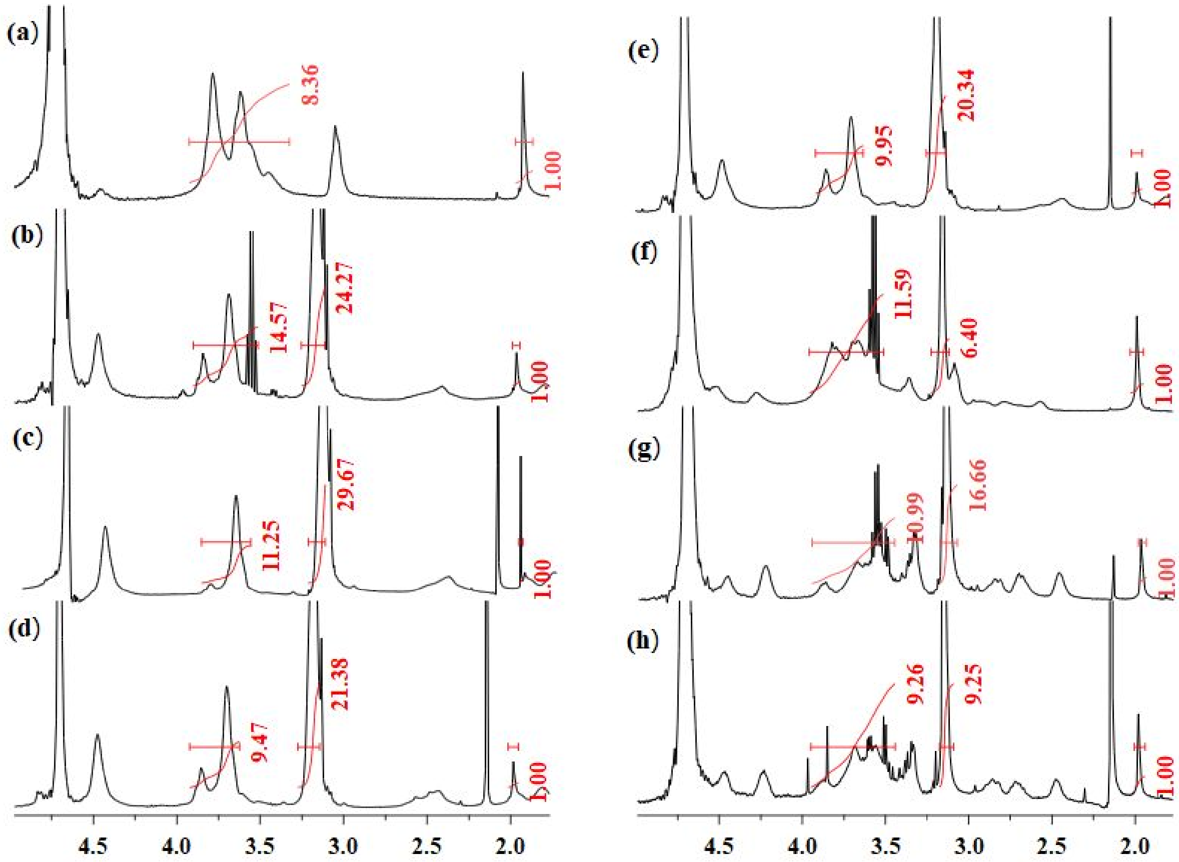

Figure S2 Integral values of chitosan derivatives, to calculate the degree of quaternization, where **(a)** is chitosan; **(b)** SQC-AETMAC; **(c)** DQC-AETMAC; **(d)** QCMC-AETMAC; **(e)** DQC-GT/AET; **(f)** SQC-GTMAC; **(g)** DQC-GTMAC; and **(h)** QCMC-GTMAC.

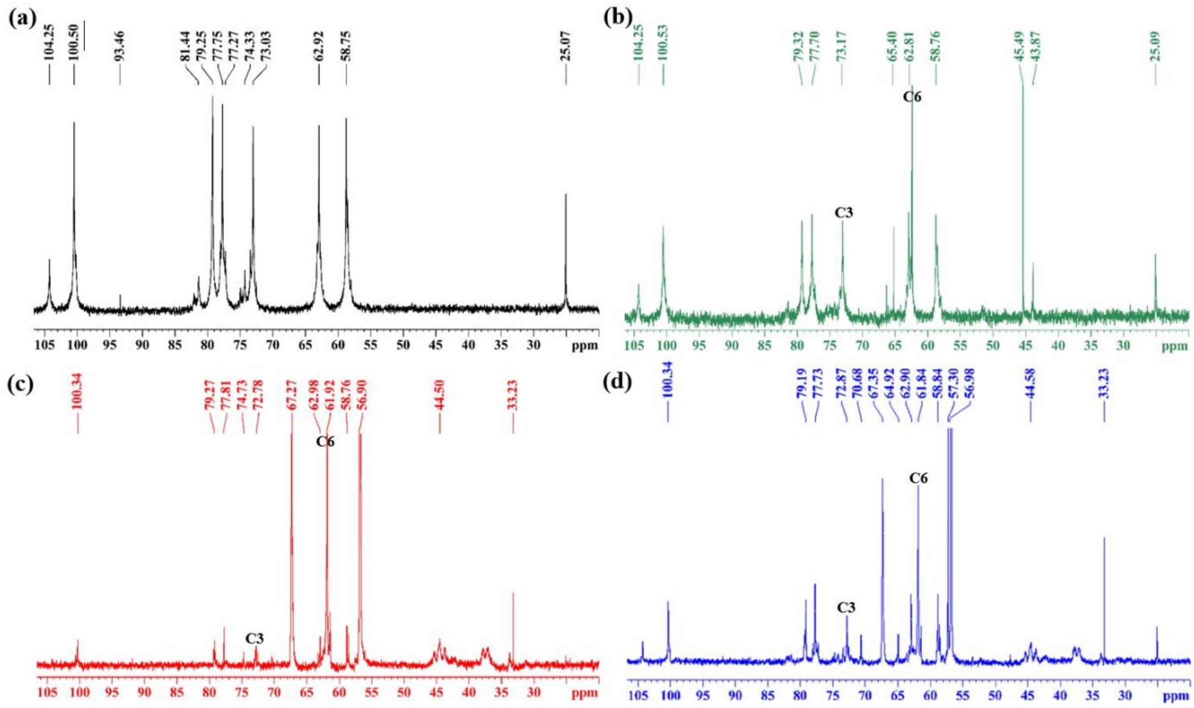

Figure S3 ^{13}C NMR spectra of **(a)** chitosan; **(b)** *O*-CMC; **(c)** DQC-AETMAC; and **(d)** DQC-GT/AET, showing differences in peak intensity for functionalisation in C-6 and C-3 position. The downfield shift for C-6 from δ=58.75 ppm (in chitosan) to approximately δ=62 ppm in *O*-CMC and other derivatives, confirming the presence of a functional group at C-6 position.

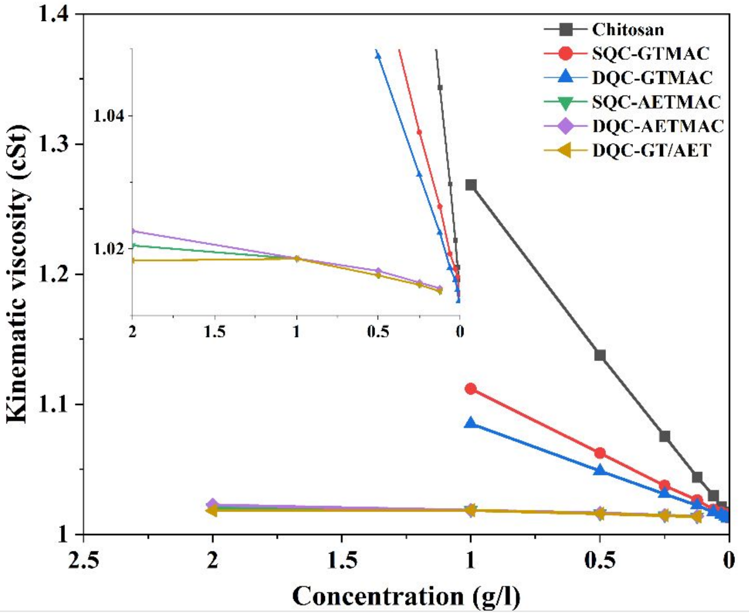

Figure S4 Graph showing kinematic viscosity of chitosan and quaternized chitosan derivatives at low concentrations, demonstrating difference in solution behavior of GTAMC- and AETMAC-functionalized materials.

```
      Phi X174_Protein F - IDSTVDIFTF YVPHRHVYGE QWIKFMKDGV NATPLPTVNT TGYIDHAAFL GTINPDTNKI 110

    MS2_Protein_Mat-A - MRAFSTLDRE NETFVPSVRV YADGETEDNS FSLKYRSNWT PGRFNSTGAK TKQWHYPSPY 60
                        SRGALSVTSI DQGAYKRSGS SWGRPYEEKA GFGFSLDARS CYSLFPVSQN LTYIEVPQNV 120
                        ANRASTEVLQ KVTQGNFNLG VALAEARSTA SQLATQTIAL VKAYTAARRG NWRQALRYLA 180
                        LNEDRKFRSK HVAGRWLELQ FGWLPLMSDI QGAYEMLTKV HLQEFLPMRA VRQVGTNIKL 240
                        DGRLSYPAAN FQTTCNISRR IVIWFYINDA RLAWLSSLGI LNPLGIVWEK VPFSFVVDWL 300
                        LPVGNMLEGL TAPVGCSYMS GTVTDVITGE SIISVDAPYG WTVERQGTAK AQISAMHRGV 360

      E. coli_Pilin_traA - MNAVLSVQGA SAPVKKKSFF SKFTRLNMLR LARAVIPAAV LMMFFPQLAM AAGSSGQDLM 60

   P. syringae_Prepilin - MNAQKGFTLI ELMIVVAIVG ILAAVAIPSY QNYAKKAAYT EVLAAMASVK TAVGVCAAQQ 60
                        GTVADCDTAA KVGVTLPSGA TTGAVNKMEI TATSAAITAT PNAFKGILTT DTCSLTPAIA 120
                        AAGSPVTWSY TGACVTNGYV KN 140

Phi 6. Spike protein p3 - MRYQGINEWL GGAKKLTTAN GEIGAIYLSA APPTDAARAD AKAVDFTAGW PSAIVDCADA 60
                        TRAKQNYLWV GDNVVHIGAK HVPLLDLWGG TGDAWQQFVG YACPMLDLCR AWGLGYASAS 120
                        VTTGSLQGYQ PSAFLDVEQQ QFAKDNLNLY GDNCLDLATS SSAQRAFLEQ CMGCALPEDC 180
                        IFGWYVKMDW EGSAVADAYA AIRVQGFATV MAPWQSVGGA GYVYARVPQK GAWMGVNLLA 240
                        YVHGTSGQPA YGIPMTLSGF TGNMGQVASK WLMLPLLMIV DPHVVQILAA LGVKRGTKSD 300
                        PRTTDVYADP KVPASRISGP MINGTVAPPA TIPATIPVPL APLGGAGGPG AQGFQVYPVF 360
                        TWGLPEFMTD VTIEGTVTAD SNGLHVVDDV RNYVWNGTAL AAIEQVNAAD GRVTLTDSER 420
                        AQLASLTVRT ASLRQQLSVG ADPLSKTSIW RRAQKADYDL LSQQIIEADT VKNLPAVTFA 480
                        QANKAAGGQS ETLWHQMYRV NDIAGDQVTA IQITGTMATG IRWSATAGGL VVDADEQDAV 540
                        IAISSGKPVK NSSDLPTADA VNYLFGITAD DMPGIVSSQK EMNSEFEEGF LQKARLWNPR 600
                        KLVENVQNAY FLMVYARDRK QFHSLVASSL AMAKLGVSTR ACKESYGC 640
```

Figure S5 Spike protein sequences of different viruses and corresponding host surface proteins. Spike proteins present on the virus surface and the receptor protein present on the surface of respective host are highlighted in red. Phi6 protein P3 interacts with pilin originating from protein prepilin. Presence of hydrophobic amino acids at the interacting domains depicts role of hydrophobic-hydrophobic interactions in initial host-virus interaction and binding.

Tables

Table S1 Virucidal activity evaluation (average $\log_{10}$ PFU) of φX174, MS2 and φ6. Viral average $\log_{10}$ reduction values of different bacteriophages exposed to different concentrations of the test compounds (50 or 100 mg/ml) for a period of 10 min under non-dried (ND) and dried (D) conditions.

		φX174		MS2		φ6	
**Test Compound**	**Conc. (mg/ml)**	**ND**	**D**	**ND**	**D**	**ND**	**D**
QCMC-AETMAC	50	6.92	6.52	3.56	2.43	8.36	8.57
	100	6.92	6.52	3.42	2.49	8.46	8.05
SQC-GTMAC	50	0.26	0.29	0.56	0.06	5.60	5.52
	100	0.29	0.28	0.6	0.03	5.60	5.52
DQC-GTMAC	50	0.39	2.73	0.79	0.65	8.57	6.63
	100	0.39	2.53	0.87	1.23	8.57	8.42

SQC-AETMAC	50	5.4	4.3	1.35	1.04	5.86	4.8
	100	6.27	4.5	1.48	1.1	6.5	5.45
DQC-AETMAC	50	4.33	6.29	2.0	1.19	7.23	6.52
	100	7.43	6.54	1.96	1.48	7.41	6.52
DQC-GT/AET	50	6.15	4.70	2.56	1.73	8.36	6.96
	100	6.66	4.79	2.62	2.07	9.5	7.45
